# Increased KL-6 levels in moderate to severe COVID-19 infection

**DOI:** 10.1371/journal.pone.0273107

**Published:** 2022-11-28

**Authors:** Maureen Cambier, Monique Henket, Anne Noelle Frix, Stéphanie Gofflot, Marie Thys, Sara Tomasetti, Anna Peired, Ingrid Struman, Anne-Françoise Rousseau, Benoît Misset, Gilles Darcis, Michel Moutschen, Renaud Louis, Makon-Sébastien Njock, Etienne Cavalier, Julien Guiot

**Affiliations:** 1 Department of Pneumology, University Hospital of Liège, Liège, Belgium; 2 Laboratory of Molecular Angiogenesis, GIGA Research Center, University of Liège, Liège, Belgium; 3 Biothèque Hospitalo-Universitaire de Liège, University Hospital of Liège, Liège, Belgium; 4 Department of Biostatistics and Medico-Economic Information, University Hospital of Liège, Liège, Belgium; 5 Department of Experimental and Clinical Medicine, Careggi University Hospital, Florence, Italy; 6 Department of Experimental and Clinical Biomedical Sciences "Mario Serio", University of Florence, Florence, Italy; 7 Department of Intensive Care, University Hospital of Liège, Liège, Belgium; 8 Department of Infectious Diseases and General Internal Medicine, Liège University Hospital, Liège, Belgium; 9 AIDS Reference Laboratory, Liège University, Liège, Belgium; 10 Fibropole Research Group, GIGA Research Center, University of Liège, University Hospital of Liège, Liège, Belgium; 11 Department of Clinical Chemistry, University of Liège, University Hospital of Liège, Liège, Belgium; Tanta University Faculty of Medicine, EGYPT

## Abstract

**Background:**

The global coronavirus disease 2019 (COVID-19) has presented significant challenges and created concerns worldwide. Besides, patients who have experienced a SARS-CoV-2 infection could present post-viral complications that can ultimately lead to pulmonary fibrosis. Serum levels of Krebs von den Lungen 6 (KL-6), high molecular weight human MUC1 mucin, are increased in the most patients with various interstitial lung damage. Since its production is raised during epithelial damages, KL-6 could be a helpful non-invasive marker to monitor COVID-19 infection and predict post-infection sequelae.

**Methods:**

We retrospectively evaluated KL-6 levels of 222 COVID-19 infected patients and 70 healthy control. Serum KL-6, fibrinogen, lactate dehydrogenase (LDH), platelet-lymphocytes ratio (PLR) levels and other biological parameters were analyzed. This retrospective study also characterized the relationships between serum KL-6 levels and pulmonary function variables.

**Results:**

Our results showed that serum KL-6 levels in COVID-19 patients were increased compared to healthy subjects (470 U/ml vs 254 U/ml, P <0.00001). ROC curve analysis enabled us to identify that KL-6 > 453.5 U/ml was associated with COVID-19 (AUC = 0.8415, P < 0.0001). KL-6 level was positively correlated with other indicators of disease severity such as fibrinogen level (r = 0.1475, P = 0.0287), LDH level (r = 0,31, P = 0,004) and PLR level (r = 0.23, P = 0.0005). However, KL-6 levels were not correlated with pulmonary function tests (r = 0.04, P = 0.69).

**Conclusions:**

KL-6 expression was correlated with several disease severity indicators. However, the association between mortality and long-term follow-up outcomes needs further investigation. More extensive trials are required to prove that KL-6 could be a marker of disease severity in COVID-19 infection.

## Introduction

A novel coronavirus, severe acute respiratory syndrome coronavirus 2 (SARS-CoV-2), appeared in Wuhan (China) in December 2019 [1, 2]. This highly pathogenic coronavirus is the causative agent for various respiratory symptoms in coronavirus disease 2019 (COVID-19) [3]. The ongoing pandemic has caused millions of deaths, resulting in a public health emergency of international concern [4]. COVID-19 diagnosis methods include computed tomography (CT), molecular tests based on nucleic acid amplification (PCR), and immunoassays [5–8]. These techniques can sometimes be expensive, time-consuming and can present a low specificity. As the pandemic rapidly spreads, there is an urgent need for fast and accurate diagnosis strategies.

Since Angiotensin-converting enzyme 2 (ACE2) is mainly expressed in the pulmonary epithelium, the SARS-CoV-2 virus can easily enter epithelial cells [9]. Thus, coronaviruses are known to induce interstitial pneumopathy and ultimately to lung fibrosis [10]. Multiple molecules are known to be associated with alveolar epithelial dysfunction and alveolar trauma [11–13]. The most described and widely used as a diagnostic and prognostic indicator in pulmonary fibrosis is Krebs von den Lungen-6 (KL-6) [11–13]. KL-6 is a mucinous high-molecular-weight glycoprotein produced by type II pneumocytes and bronchial epithelial cells encoded by the MUC1 gene. In normal lungs, this protein is mainly involved in lung fibroblasts’ migration, proliferation, and survival [14, 15]. Interestingly, KL-6 production is reported to be increased during epithelial lesions and cellular regeneration [16–18]. In COVID-19 infection, KL-6 serum levels could be interesting for diagnosis, prognosis, and therapeutic response evaluation. Previously, we have shown that KL-6 is associated with ILD severity in COVID-19 infection [5]. Indeed, we demonstrated that high KL-6 levels could be linked to oxygenation levels and other indicators of disease severity. Our present study aims to confirm and characterize more deeply our previously described results with a more extensive retrospective cohort.

## Materials and methods

### Cohort characteristics

Our study retrospectively compared KL-6 levels between a cohort of 222 infected patients (COVID-19 PCR positive patients hospitalized in Liège University Hospital between April 25^th^, 2020 to February 25^th^, 2021) and a reference group. The reference group was composed of 70 healthy subjects (HS). Samples from HS dated before the SARS-CoV-2 pandemic. Criteria to describe moderate and severe cases of COVID-19 were used as recommended by the WHO Organization (WHO-2019-nCoV-clinical-2021-2) COVID-19 disease severity categorization. Patients with clinical signs of pneumonia (fever, cough, dyspnea, fast breathing) but no signs of severe pneumonia, including SpO2 ≥ 90% on room air, are categorized as a moderate disease. Patients with clinical signs of pneumonia (fever, cough, dyspnea) plus one of the following: respiratory rate > 30 breaths/min; severe respiratory distress; or SpO2 < 90% on room air were considered severe diseases. Demographical (including age, sex, past medical history), clinical (including oxygen levels, Intensive Care Unit (ICU) indication), and admission laboratory indexes (including serum CRP, serum KL-6, serum LDH, complete blood count, and fibrinogen) were collected for infected patients. For the reference group, demographical and serum CRP, serum KL-6, complete blood count, and fibrinogen data were collected (Table 1).

**Table 1:** Demographic and clinical characteristics of HS and COVID-19 patients.

	HS (n = 70)	COVID-19 (n = 222)	P value
**Gender, M (%)**	35 (50%)	154 (69,3%)	0.004
**Age**	58 (52–64)	67 (57–75)	<0.00001
**Leukocytes (ml)**	6,21 (5,13–7,43)	8,01 (5,47–10,21)	0.0005
**Neutrophils**			
%	55 (49–61)	81 (72–87)	<0.00001
number/ml	3,38 (2,81–4,22)	6,29 (4,13–8,61)	<0.00001
**Lymphocytes**			
%	33 (29–38)	11 (6–18)	<0.00001
number/ml	2,2 (1,71–2,49)	0,81 (0,54–1,22)	<0.00001
**Monocytes**			
%	7,65 (6,7–8,9)	5,45 (3,6–8)	<0.00001
number/ml	0,48 (0,39–0,62)	0,42 (0,25–0,69)	0.28
**Eosinophils**			
%	0,13 (0,09–0,23)	0,01 (0–0,05)	<0.00001
number/ml	2,2 (1,6–3,8)	0,1 (0–0,7)	<0.00001
**Basophils**			
%	0,5 (0,3–0,7)	0,2 (0,1–0,4)	<0.00001
number/ml	0,03 (0,02–0,05)	0,02 (0,01–0,03)	<0.00001
**CRP (mg/L)**	1 (0,5–2,4)	87 (42–157)	<0.00001
**Fibrinogen (g/L)**	2,88 (2,56–3,43)	5,48 (4,43–6,95)	<0.00001
**LDH (U/L)**		387 (277–533)	
**PLR**		291,30 (184,9–456,9)	
**KL-6 (U/ml)**	254 (191–308)	470 (330–738)	<0.0001
**SPO**_**2**_ **(%)**		90 (85–95)	
**ICU, Yes (%)**		123 (55.4%)	
**RI, Yes (%)**		62 (27.9%)	
**Dead, Yes (%)**		68 (30.6%)	

HS, Healthy Subjects; COVID-19, coronavirus disease 2019; CRP, C-Reactive Protein; ICU, Intensive care unit; IQR, Inter Quartile Range; KL-6, Krebs von den Lungen 6; M, Male; RI, Respiratory Insufficiency; LDH, lactate dehydrogenase; PLR, Platelet/Lymphocytes Ration; SPO_2_, Oxygen saturation. Data following a normal distribution are expressed as mean (+/- SD); otherwise, they are expressed as median (+/- IQR).

### Ethical considerations

The ethics committee approved the protocol of the University Hospital of Liège (Belgian Number: B707201422832; ref: 2021/89).

### Biomarker measurements

We retrospectively extracted from the medical file levels of KL-6 as marker of pulmonary fibrosis). Fibrinogen (a coagulopathy marker [19, 20]), lactate dehydrogenase (LDH) (a lung injury marker [18, 19, 21]), C-reactive protein (CRP) (an inflammatory marker) and platelet/lymphocytes ratio (PLR) (a promising prognostic marker of COVID-19 severity [22]) were also assessed. Pulse oximetry (SPO_2_) was monitored during patients’ hospitalization to assess the respiratory status on day 7 post-SARS-CoV-2 detection. Respiratory insufficiency was defined as lower than 90%. Blood samples were collected in BD Vacutainer SST II Advance tubes and centrifuged to quantify LDH, CRP, and KL-6. The reference values for LDH and CRP on serum are respectively between 125–220 U/L and 0–5 mg/L. For KL-6 measurement, we used a chemiluminescent light immunoassay on the Fujirebio Lumipulse G1200 instrument (Tokyo, Japan). The threshold for high levels of KL-6 was 453.5 U/ml. Other analyses were run on the Abbott Alinity platform (Abbott Park, IL, USA). Blood samples were collected in citrate tubes for fibrinogen measurement, and a coagulometric test was made using Thrombin reagent (Siemens, Germany). The reference values for KL-6 on serum are between 118–627 U/ml.

### Statistical analysis

Normality was verified using the Kolmogorov-Smirnov test. The contingency tables were analyzed with the *Fisher test*. When the data follow a normal distribution, the results are expressed as mean (± standard deviation (SD)) and analyzed with an unpaired *student’s T test*. Otherwise, they are expressed as the median (interquartile range (IQR)) and analyzed with the Mann-Whitney test to compare two groups. The performance of the KL-6 to differentiate patient COVID-19 from no COVID-19 was assessed by constructing receiver operating characteristic (ROC) curves. *p* values < 0.05 were considered as statistically significant. Data are analyzed with Graph pad PRISM® version 8 software.

## Results

### Study population

**Fig 1:**
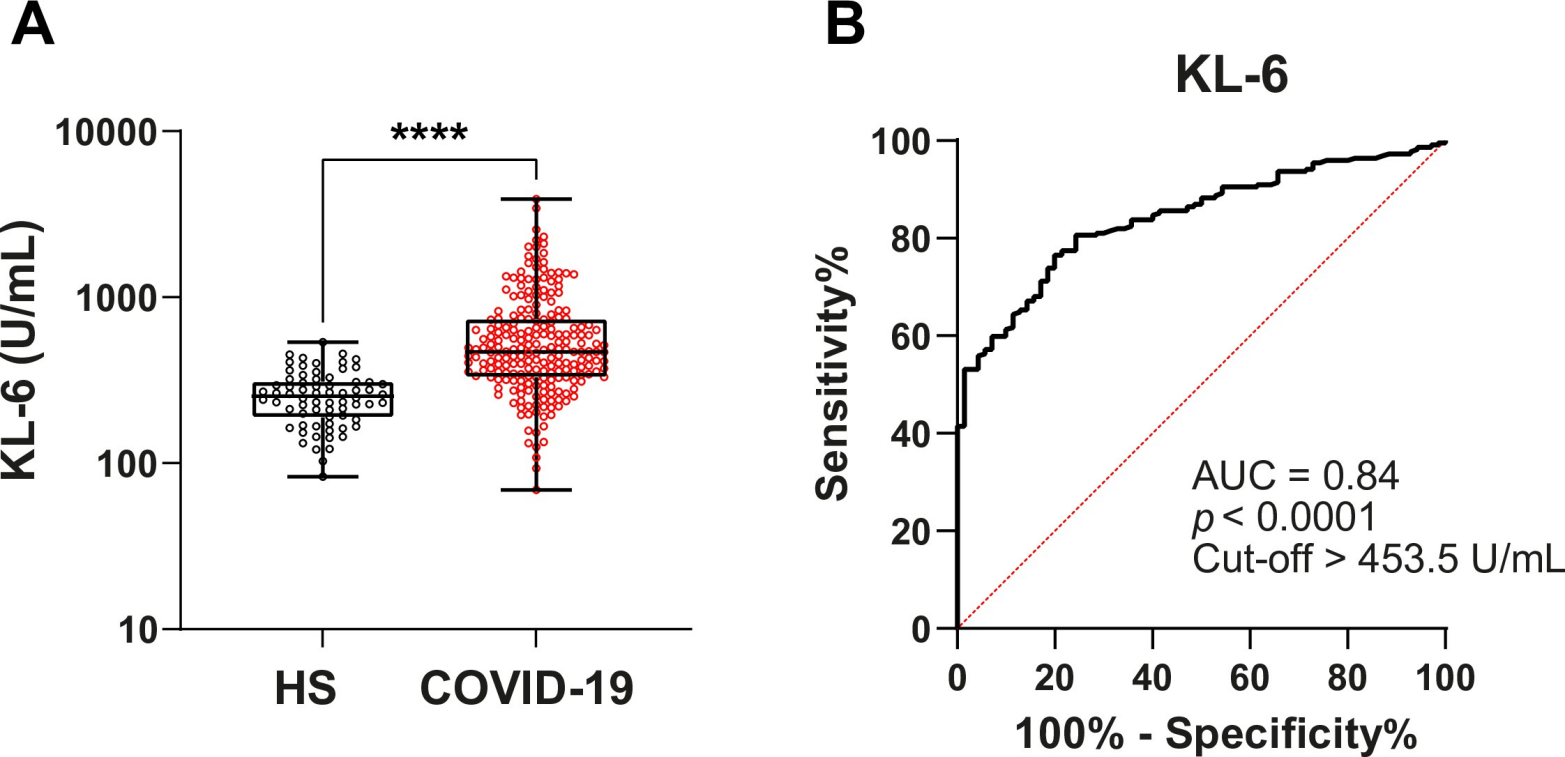
Capacity of KL-6 to discriminate between COVID-19 patients and HS. (**A**) KL-6 level in HS and in COVID-19 patients. Data are non-normally distributed and are analyzed using a non-parametric two-tailed Mann-Whitney test. ****P < 0.00001. (**B**) ROC curve analysis to determine the level of KL-6 which will enable to discriminate COVID-19 patients and our reference population (HS).

COVID-19 patient characteristics are listed in Table 1. The median age of COVID-19 patients was 67 years old with a male predominance (69.3%). Our groups were not fully matched concerning age and gender. Therefore, we performed a specific analysis in the COVID group in which we did not find any correlation between KL-6, age and gender (data not shown). We compared the levels of different serum biomarkers associated with lung damage between HS and COVID-19 groups. Interestingly, COVID-19 patients present higher levels of KL-6 (470 U/ml *vs* 254 U/ml, P <0.00001). Fibrinogen (5.48 g/L vs 2.88 g/L, P <0.00001) and CRP (87 mg/L vs 1 mg/L, P <0.00001) are also increased in COVID-19 patients compared to HS (Table 1 and Fig 1A).

### Capacity of KL-6 to discriminate between COVID-19 subjects and HS

ROC curve analysis enabled us to identify that the KL-6 level of 453.5 U/ml is the cut-off value for the discrimination of COVID-19 patients and HS (area under the ROC curve (AUC) = 0.84 at 53% sensibility and 97% specificity, P < 0.0001) (Fig 1B). Indeed, serum level of KL-6 ≥ 453.5 U/ml was associated with COVID-19 disease.

### Correlation between KL-6 levels and markers of COVID-19 severity

**Fig 2:**
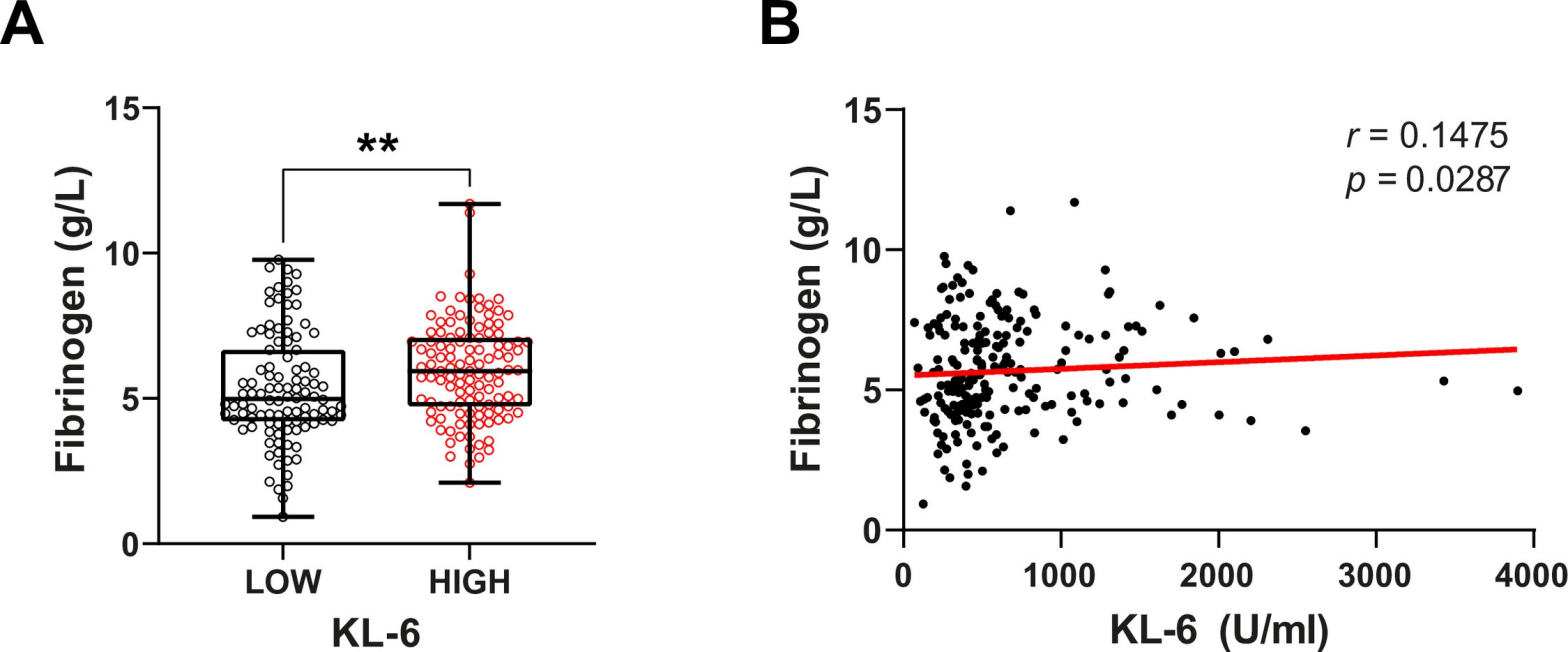
Correlation between KL-6 and Fibrinogen levels in COVID-19 patients. (**A**) Comparison of Fibrinogen levels between COVID-19 patients with high and low KL-6 levels. High KL-6 patients display a significantly higher Fibrinogen level. Data are analyzed using a non-parametric two-tailed Mann-Whitney test. **P < 0.01. (**B**) Correlation between KL-6 and Fibrinogen levels. Data are analyzed using Spearman correlation.

**Fig 3:**
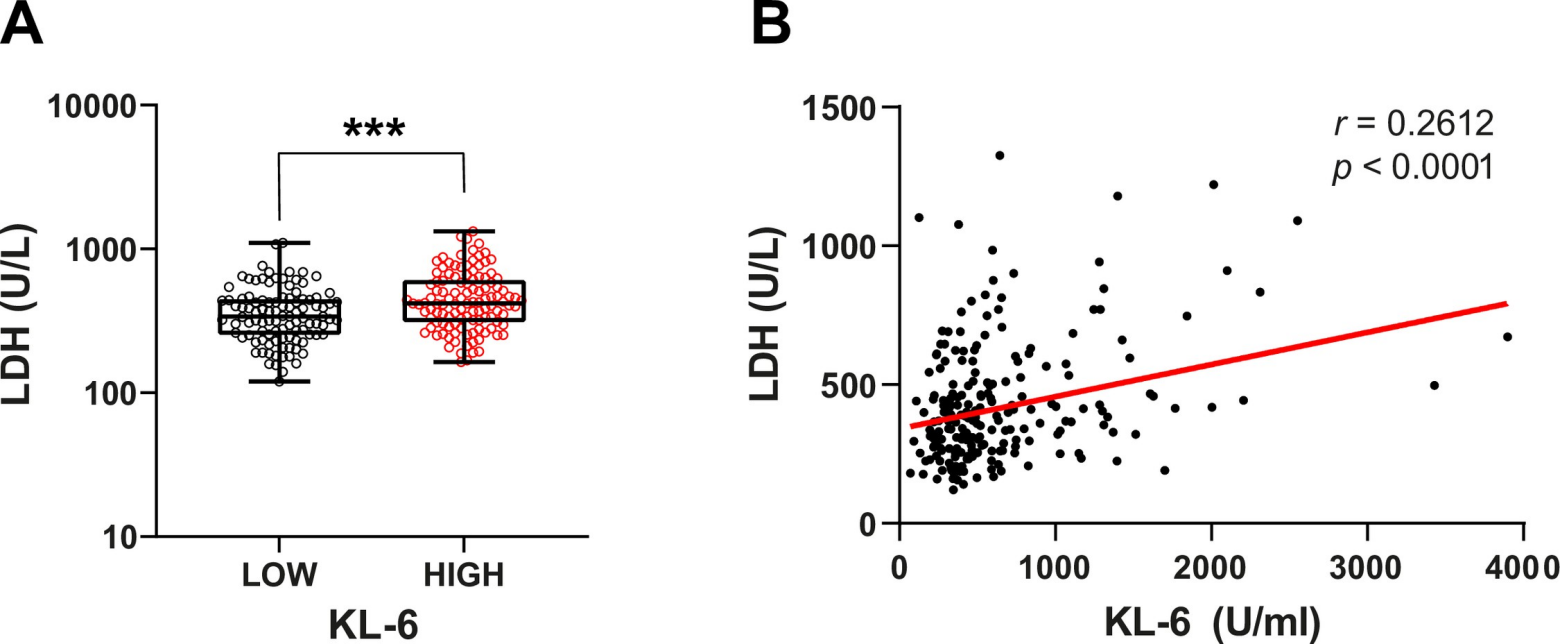
Correlation between KL-6 and LDH levels in COVID-19 patients. (**A**) Comparison of LDH levels between COVID-19 patients with high and low KL-6 levels. High KL-6 patients display a significantly higher LDH level. Data are analyzed using a non-parametric two-tailed Mann-Whitney test. ***P < 0.001. (**B**) Correlation between KL-6 and LDH levels. Data are analyzed using Spearman correlation.

**Fig 4:**
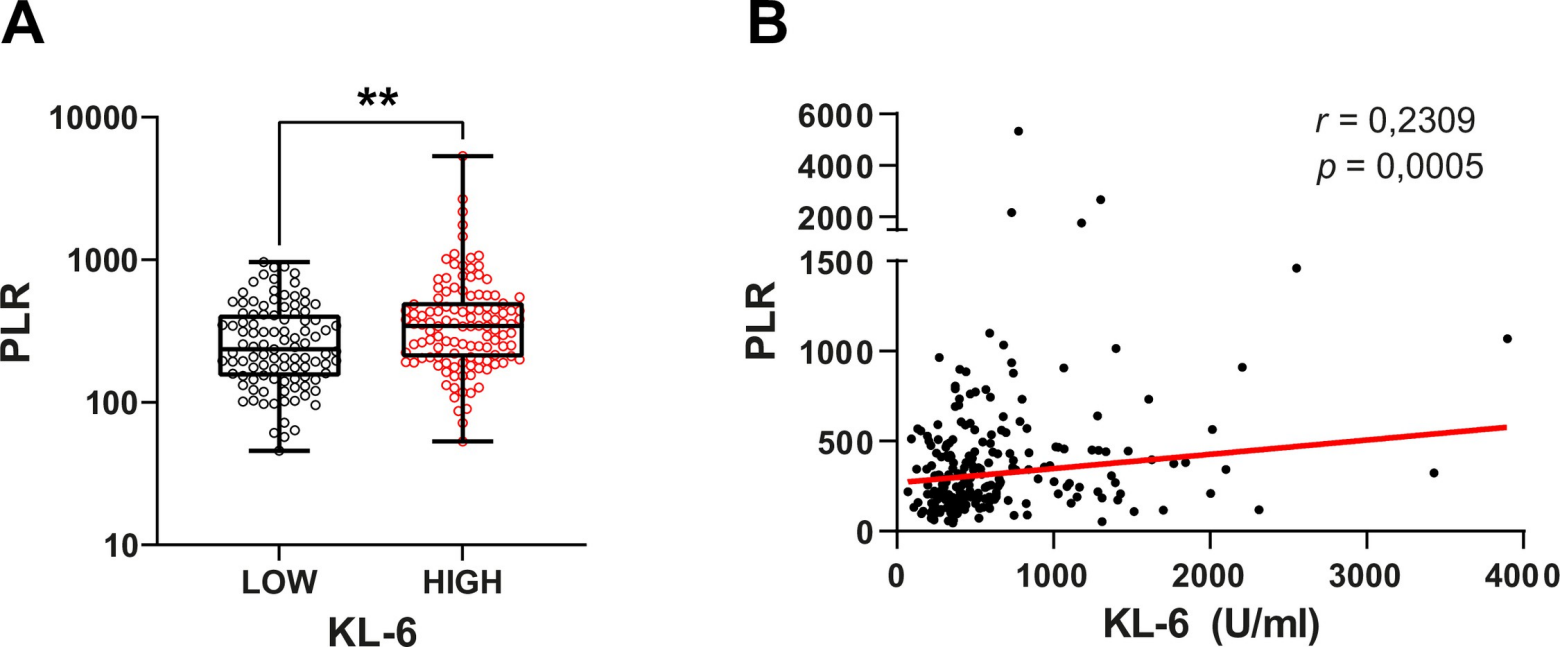
Correlation between KL-6 and PLR levels in COVID-19 patients. (**A**) Comparison of PLR levels between COVID-19 patients with high and low KL-6 levels. High KL-6 patients display a significantly higher PLR levels. Data are analyzed using a non-parametric two-tailed Mann-Whitney test. **P < 0.01. (**B**) Correlation between KL-6 and PLR levels. Data are analyzed using Spearman correlation.

The correlation between the expression level of serum KL-6 and several indicators of disease severity (fibrinogen, LDH, PLR) was studied in the group of COVID-19 patients. Serum level of KL-6 is positively correlated with fibrinogen level (r = 0.14, P = 0.0287) (Fig 2A and 2B), LDH level (r = 0,26, P < 0.0001) (Fig 3A and 3B) and PLR level (r = 0.23, P = 0.0005) (Fig 4A and 4B). All these observations suggest that serum levels of KL-6 could be associated with COVID-19 severity. However, high levels of KL-6 do not seem correlated to CRP levels (r = 0.04, P = 0.48) (S1A Fig) We did not find any correlation between CRP levels and serum level of KL-6 in COVID-19 patients (S1B Fig) (P = 0.34).

### Correlation between KL-6 levels and respiratory status

We did not find any correlation between KL-6 and SPO_2_ levels (r = 0.04, P = 0.69, S2 Fig). A significant proportion of the COVID-19 cohort (103/222) experienced an ICU admission. However, high KL6 levels do not seem to be linked to ICU admission (P = 0.69, S3A Fig), nor with respiratory insufficiency (RI) (P = 0.85, S3B Fig).

## Discussion

As the SARS-CoV-2 pandemic is evolving, new biomarker identification will play a role in the precise and early diagnosis of COVID-19 disease. Indeed, the specificity of a standard PCR testing only approaches 80% sensitivity and 98–99% specificity [23]. Moreover, some patients can still present radiological abnormalities with a negative test. Since KL-6 is a recognized indicator of lung fibrosing process, it may be help predict the outcome of these patients and identify ILD.

As the main target of SARS-CoV-2 is the lung epithelium, SARS-CoV-2 infection results in the secretion of proinflammatory cytokines known to damage alveolar epithelial cells [24]. Ultimately, this inflammatory reaction causes the release of KL-6 in the blood flow. Thus, KL-6 constitutes a specific biomarker of damaged lung type II pneumocytes [17]. The mucoprotein KL-6 has been several times proposed as a promising biomarker for detecting intertitial lung diseases such as idiopathic pulmonary fibrosis. It is also demonstrated as a prognostic biomarker for the severity of acute respiratory distress syndrome (ARDS) [25, 26].

In this study, we appraised the potential use of KL-6 as a prognostic value to predict lung disease in COVID-19 patients. We identified a cut-off of KL-6 levels to discriminate healthy subjects from COVID-19 patients. We detected an increase of KL-6 serum levels of COVID-19 patients (470 U/ml vs. 254 U/ml, P <0.00001) as in many other studies [17, 27, 28]. Interestingly, the KL-6 level was positively correlated with the expression of several biological features which are well-known predictors of severe COVID-19 outcomes, such as fibrinogen, PLR, and LDH. Indeed, one factor of coagulopathy observed in patients hospitalized with COVID-19 is characterized by elevations in fibrinogen [29]. We observed the same pattern in our COVID-19 cohort (COVID-19 5.48 g/L vs HS 2.88 g/L, P<0.00001). Moreover, fibrinogen is secreted by hepatocytes but also by airway epithelial cells [30]. As part of inflammatory responses, it is also increased in subjects with acute severe asthma [31]. We found a positive correlation between KL-6 and fibrinogen, suggesting a possible link between KL-6 and worst patient conditions.

The virus-cell interaction in epithelial cells leads to the activation of hyper-inflammatory responses by promoting of IL-6 trans-signaling [32]. Thus, PLR was used as an indicator reflecting an indirectly inflammatory state. In our study, patients with elevated PLR showed increased KL-6 expression, as mentioned in other studies [5, 33, 34].

We also found a positive correlation between LDH and KL-6. Since LDH is present in lung tissue, detecting higher amount of this enzyme in the circulation during lung damaged pathologies is not surprising. It is associated with the worst outcomes in Severe Acute Respiratory Syndrome [35, 36].

As defined by median SpO_2_ at admission, we previously showed that high KL-6 levels were significantly related to lung disease severity [5]. With a larger cohort of COVID-19 patients, SpO_2_ levels no longer seem related to high KL-6 levels anymore. It can be explained by the recent use of dexamethasone since the publication of the Recovery trial modifying the clinical evolution of COVID-19 infected patients [37]. Indeed, dexamethasone can reduce lung inflammation in the early phase and diffuse alveolar damage that can modify biomarkers levels and their significance level. Of course, we assume that many other factors have to be considered. Indeed, the pulmonary system is damaged during SARS-CoV-2 infection but also the cardiovascular system.

Regarding the other indicators of disease severity investigated, increased KL-6 levels were not associated with dyspnea severity or ICU admission. In a recent study published by Scotto *et al*. [38], KL-6 levels were used as a predictive value for mortality at the time of patient enrolment. We could not find a correlation in our retrospective evaluation, but we assume that many other factors must be considered. Still, high KL-6 levels were interestingly correlated with other acute phase parameters suggesting a link with the COVID-19-associated inflammatory response.

## Conclusions

In this study, we retrospectively established a serum KL-6 cut-off value that discriminates healthy subjects from COVID-19 infected patients. We appraised the pathogenic relevance of KL-6 expression through correlations with several disease severity biomarkers. However, the association with mortality and other severity parameters needs further investigation with a measurement in the early infection phase.

## Supporting information

S1 Fig Correlation between KL-6 and CRP levels in COVID-19 patients. (**A**) Comparison of CRP levels between COVID-19 patients with high and low KL-6 levels. Data are analyzed using a non-parametric two-tailed Mann-Whitney test. *ns*: not significant. (**B**) Correlation between KL-6 and CRP levels. Data are analyzed using Spearman correlation.
(TIF)

S2 Fig Correlation between KL-6 and SpO_2_ levels in COVID-19 patients. (**A**) Comparison of SpO_2_ levels between COVID-19 patients with high and low KL-6 levels. Data are analyzed using a non-parametric two-tailed Mann-Whitney test. *ns*: not significant. (**B**) Correlation between KL-6 and SpO_2_ levels. Data are analyzed using Spearman correlation.
(TIF)

S3 Fig Multi-panel describing results. (**A**). Comparison of KL-6 levels between patients with COVID-19 admitted to intensive care (ICU) or not. (**B**) Comparison of KL-6 levels between patients with COVID-19 with respiratory insufficiency. *ns*: not significant. Data are analyzed using a non-parametric two-tailed Mann-Whitney test.
(TIF)
