# Supplementary figures and images for: Increased KL-6 levels in moderate to severe COVID-19 infection
Source: PLoS One. 2022 Nov 28;17(11):e0273107. doi: 10.1371/journal.pone.0273107 (PMC9704627; doi:10.1371/journal.pone.0273107)

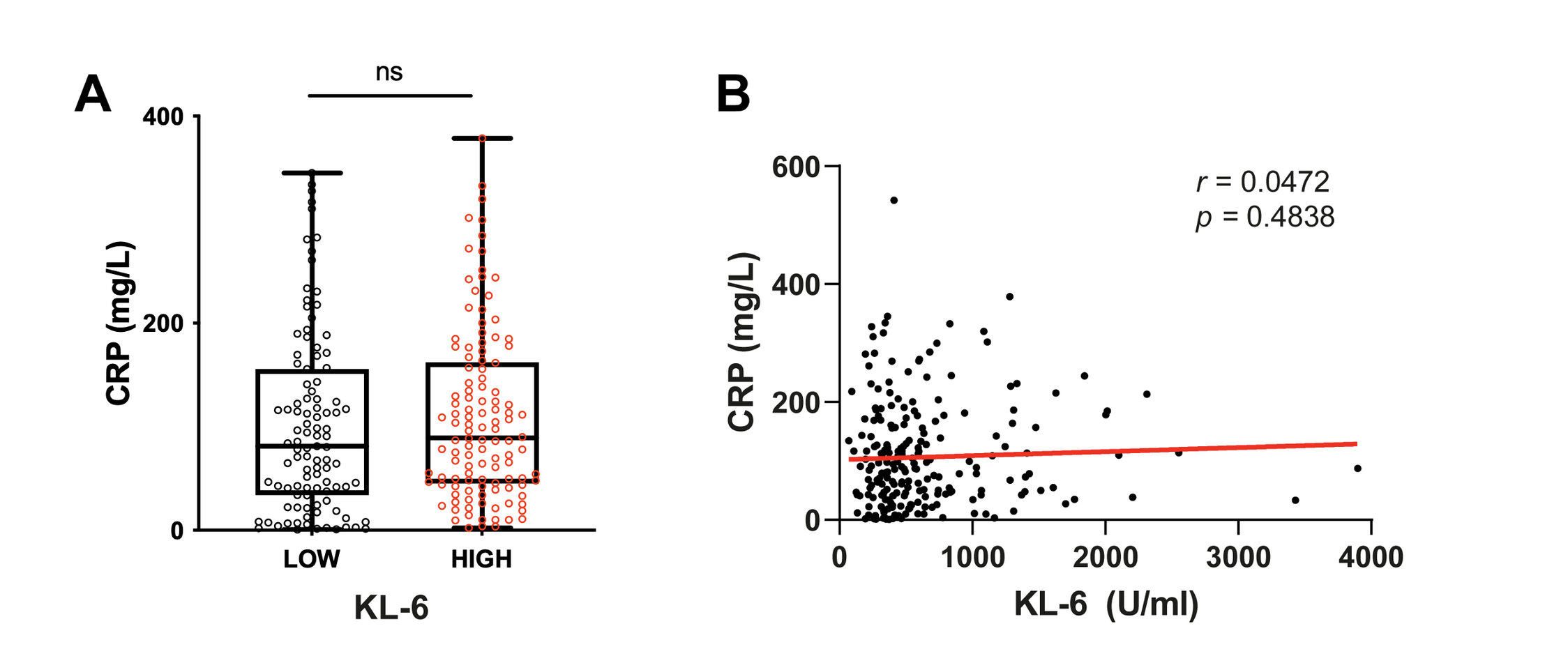

Supplement: S1 Fig — (A) Comparison of CRP levels between COVID-19 patients with high and low KL-6 levels. Data are analyzed using a non-parametric two-tailed Mann-Whitney test. ns: not significant. (B) Correlation between KL-6 and CRP levels. Data are analyzed using Spearman correlation. (TIF) [file pone.0273107.s001.tif]

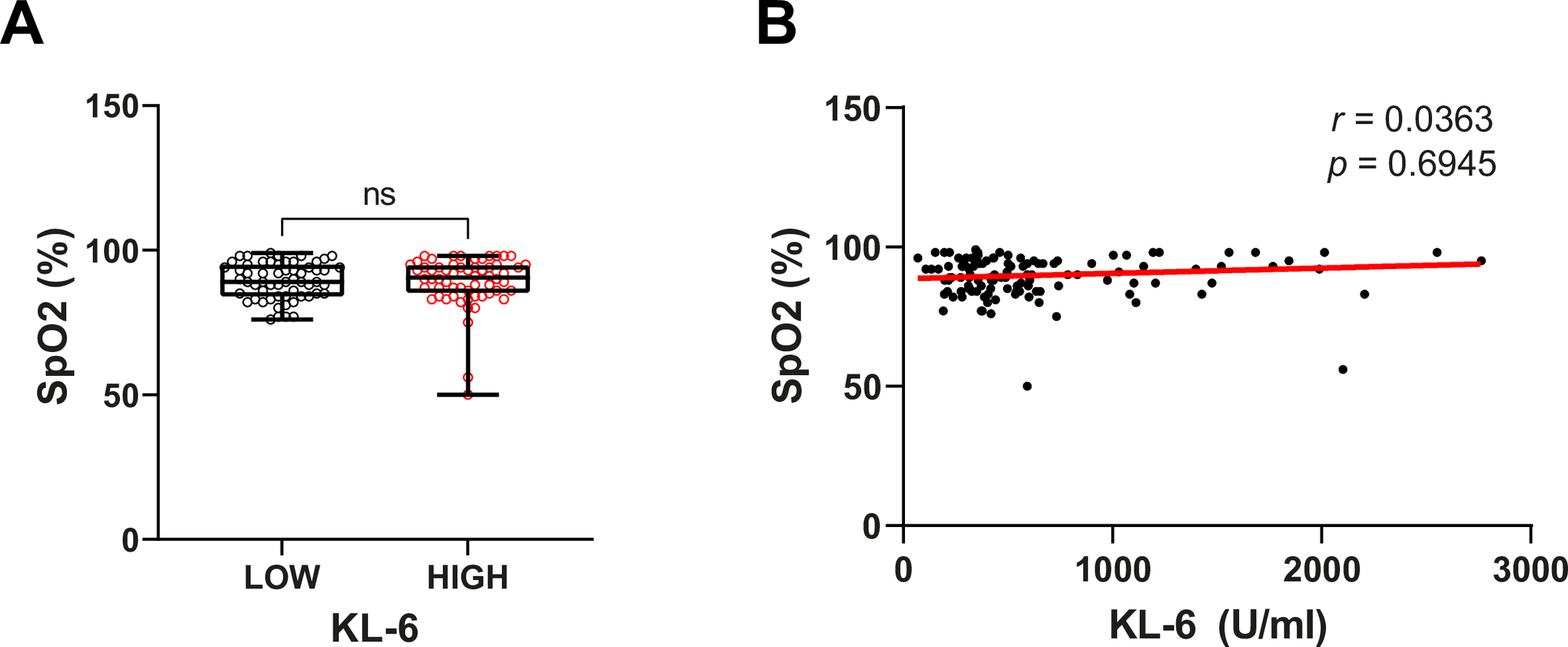

Supplement: S2 Fig — (A) Comparison of SpO2 levels between COVID-19 patients with high and low KL-6 levels. Data are analyzed using a non-parametric two-tailed Mann-Whitney test. ns: not significant. (B) Correlation between KL-6 and SpO2 levels. Data are analyzed using Spearman correlation. (TIF) [file pone.0273107.s002.tif]

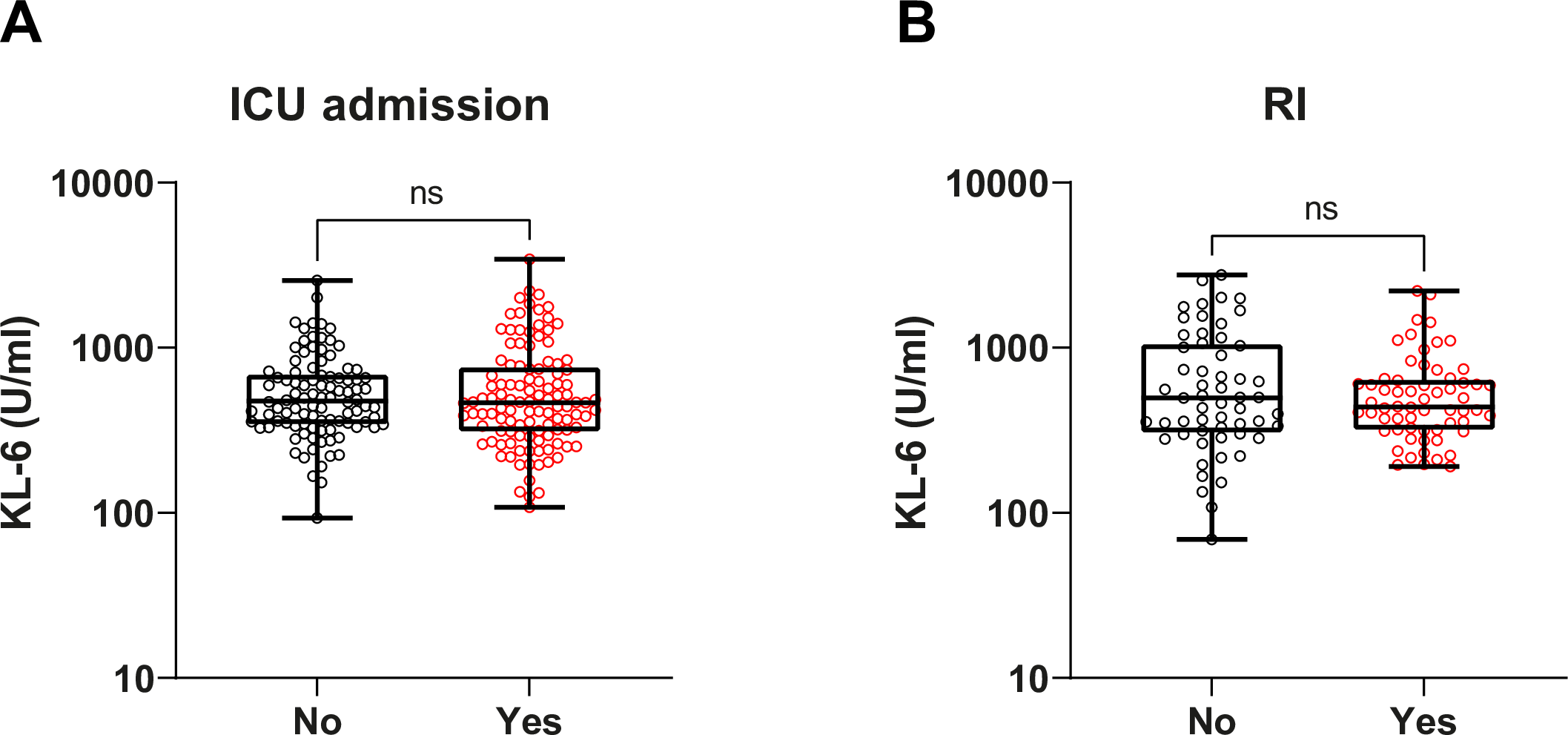

Supplement: S3 Fig — (A). Comparison of KL-6 levels between patients with COVID-19 admitted to intensive care (ICU) or not. (B) Comparison of KL-6 levels between patients with COVID-19 with respiratory insufficiency. ns: not significant. Data are analyzed using a non-parametric two-tailed Mann-Whitney test. (TIF) [file pone.0273107.s003.tif]
